# 

**Published:** 1893-11

**Authors:** 


### CONTENTS.

### EDITORIAL.

PAGE

Isopathy in the Dominant School, . . . . . . . . 513

### MISCELLANEOUS.

Principles Involved in the Law of Cure. W. E. Ledyard, M. D., . 515

Furuncles, . . . . . . . . . . . 525

Apoplexy, . . . . . . . . . . . 525

Provings and Clinical Observations with High Potencies. M. Macfarlan, M. D., . . . . . . . . . . 526

Potentiation Physiologically Proven. Prof. Dr. Gustav Jaeger. Translated by B. Fincke, M. D., . . . . . . 535

Homœopathy in Animals. Proceedings of I. H. A., . . . . 545

Siftings Saved. I. Dever, M. D., . . . . . . . 548

Gleanings. F. H. Lutze, M. D., . . . . . . . . 551

Accidental Proving of Carbolic Acid. J. C. Fahnstock, M. D., . 554

American Institute of Homœopathy. Wm. E. Leonard, M. D., . 555

The Relation of Pain to Swallowing. A McNeil, M. D., . . . 556

In Memoriam. Dr. Samuel Swan, . . . . . . . 557

### BOOK NOTICES.

Roger's Homœopathic Guide, . . . . . . . . 560

### NOTES AND NOTICES.

Dr. A. Quackenbush—Dr. F. H. Krebs—Fun for Doctors, . . . 560

